# In Vitro Chromosome Translocations: Modeling Critical Drivers of Human Neoplasms

**DOI:** 10.3390/cimb48101032

**Published:** 2026-10-04

**Authors:** Nuray Tynyshtykbayeva, Kamilla Varga, Szilvia Lukacsi, Zsolt Fabian

**Affiliations:** 1 Faculty of Science, Institute of Biology, Eötvös Loránd University, 1 Pázmány Péter Sétány, 1117 Budapest, Hungary; nuray@student.elte.hu; 2 Translocon Biotechnologies PLC, 6. Akadémia str., 1054 Budapest, Hungary; kamilla.vogel_varga.dr@translocon.hu (K.V.); szilvia.lukacsi@translocon.hu (S.L.); 3 Faculty of Clinical and Biomedical Sciences, School of Medicine and Dentistry, University of Lancashire, Fylde Rd, Preston PR1 2HE, UK

**Keywords:** chromosome translocation, double-strand DNA break, DNA repair, reciprocal translocations, meganucleases, engineered nucleases, CRISPR/Cas9, TIGR

## Abstract

Chromosomal abnormalities are among the most common molecular characteristics of human cancer cells and are believed to play an essential role in the genesis of tumor driver molecules. Chromosome translocations are one of the first recognized tumor-related chromatin alterations; many of them are so typical to certain neoplasms that they are used as diagnostic biomarkers in contemporary clinical histopathology. Understanding their molecular roles in carcinogenesis is essential to the development of novel targeted therapeutics but this has been hindered by the difficulties in establishing appropriate in vitro models. Here, we review the recent advances of the field covering its historical background, medical relevance and most recent technological breakthroughs in chromosomal translocation modeling that may facilitate the innovations of related tumor-agnostic therapeutic modalities.

## 1. Introduction

Since Walther Flemming’s initial description of chromosomes as thread-like structures in 1882, the field of cytogenetics has evolved into a multi-dimensional study of chromatin organization and its pathological disruptions [1]. Chromosomes are now recognized as a fundamental organizational units of the genetic material in eukaryotic cells, rendering chromatin into discrete physical assemblies affecting all key chromatin functions from transcription through replication to chromosome recombination and segregation of genetic information during cell division [2]. In the human chromatin, histone-associated DNA is organized into 23 pairs of homologous chromosomes that practically employ the whole nucleoplasm [3,4,5]. The karyotypic arrangement is founded upon the principle of homology, so that—although, they may differ at the level of nucleotide sequence increasing genetic diversity—each pair member carries identical gene *loci* [4,6].

**Figure 1:**
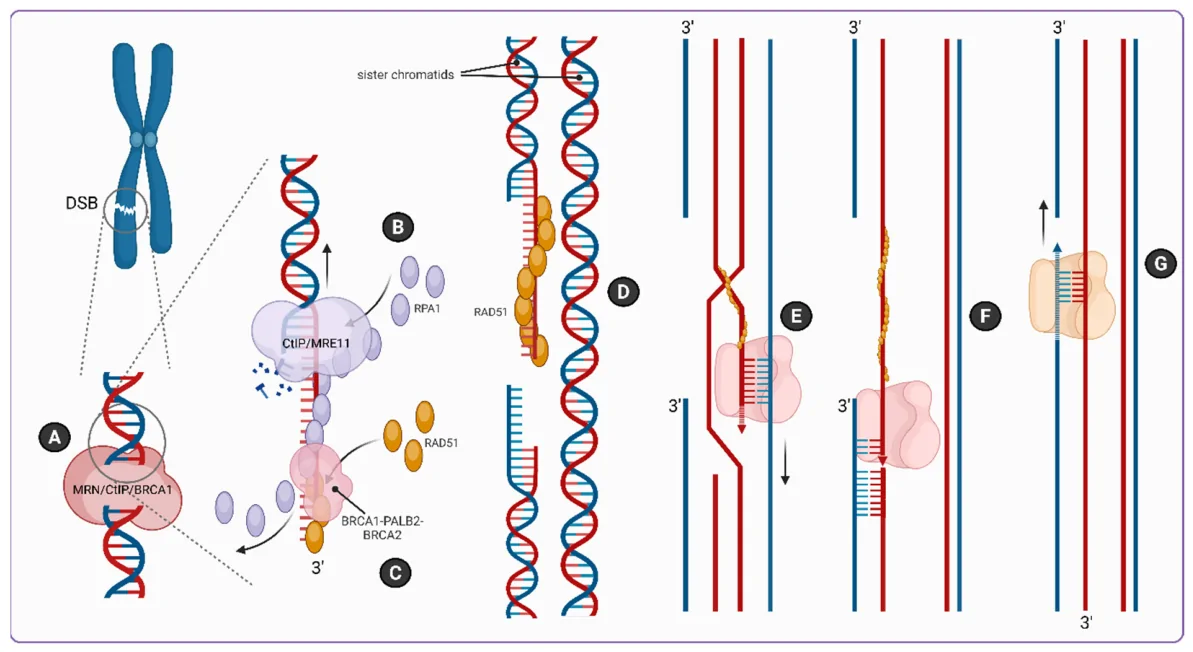
Somatic double-strand DNA break reparation by homology-directed repair (HDR). HDR begins with the recruitment of the damage recognition complex containing the MRN, CtIP and BRCA1 proteins to the damaged locus (**A**). The critical pathway-choice step upon DSBs is the CtIP/MRE11-mediated resection of the free DNA ends (**B**), that bias DSB repair toward HDR. Temporarily, overhanging single-stranded DNAs (ssDNA) are protected by RPA1 that is soon exchanged to RAD51 by the BRCA1-cooperating PALB2-BRCA2 complex (**C**). RAD51-binding allows precise alignment of the ssDNA to its homologous counterpart (**D**) and strand invasion (**E**) followed by the repair synthesis from a homologous template (**E**). If a double Holliday junction is not formed, termination of the synthesis from the homologous template resolves the D-loop (**F**) allowing complementary strand synthesis using the repaired strand as template (synthesis-dependent strand annealing) (**G**). Straight black arrows in steps B, E and G indicate the direction of the movement of the enzymes depicted.

On one hand, this homology allows genetic diversity through the reciprocal exchange of DNA between homologous chromosomes during prophase I of meiosis [7,8]. The molecular initiation of crossing over begins with DNA double-strand breaks (DSBs) at specific recombination hotspots, typically mediated by the SPO11 protein or its direct orthologs [8]. DSBs do not occur in isolation but are localized within chromatin loops, that are tethered to a proteinaceous structural axis [9]. As the progression of meiosis, the axes of homologous chromosomes align and synapse to form the synaptonemal complex (SC), a ladder-like assembly that facilitates the search for homology and stabilizes chromosome pairing [10,11]. Homology between chromosomes, however, also allows high-fidelity repair of a damaged chromosome—the process termed homology-directed repair (HDR)—using homologous DNA as a template to restore sequences at the DSB locus [12,13,14]. This process involves the CtIP and Dna2 for the 5′ to 3′ resection of the free DNA ends at DSB *loci* that results in the formation of overhanging 3′ single-stranded DNA (ssDNA) strands, a necessary prerequisite for RPA and RAD51 binding (Figure 1) [13].

Following the latter one, homologous *loci* align and form the so-called synapsis, where DMC1 and RAD51 facilitate invasion of the overhanging 3′ single-stranded DNA into the intact homologous locus leading to the formation of a Holliday junction (HJ) [15]. Upon the meiotic crossing over, programmed DSBs are repaired preferentially using homologous DNA strands rather than the sister chromatids, leading to the physical interlocking of homologs through chiasmata following the formation of double Holliday junctions (dHJs) [13,15,16]. The resolution of dHJs into crossovers also provides the tension necessary for chromosomes to resist the poleward forces of the meiotic spindle, a critical requirement of preventing nondisjunction and consequent aneuploidy [17]. During the HDR, however, sister chromatids are preferred to serve the sequence information for the reparation of somatic DSBs primarily by skipping the formation of dHJs [18]. Indeed, HDR prefers dissolution of the D-loop upon termination of the repair synthesis of the invading strand and complete synthesis of the complementary strand of the damaged DNA using the repaired strand as a template, a process termed synthesis-dependent strand annealing (Figure 1) [19,20].

**Figure 2:**
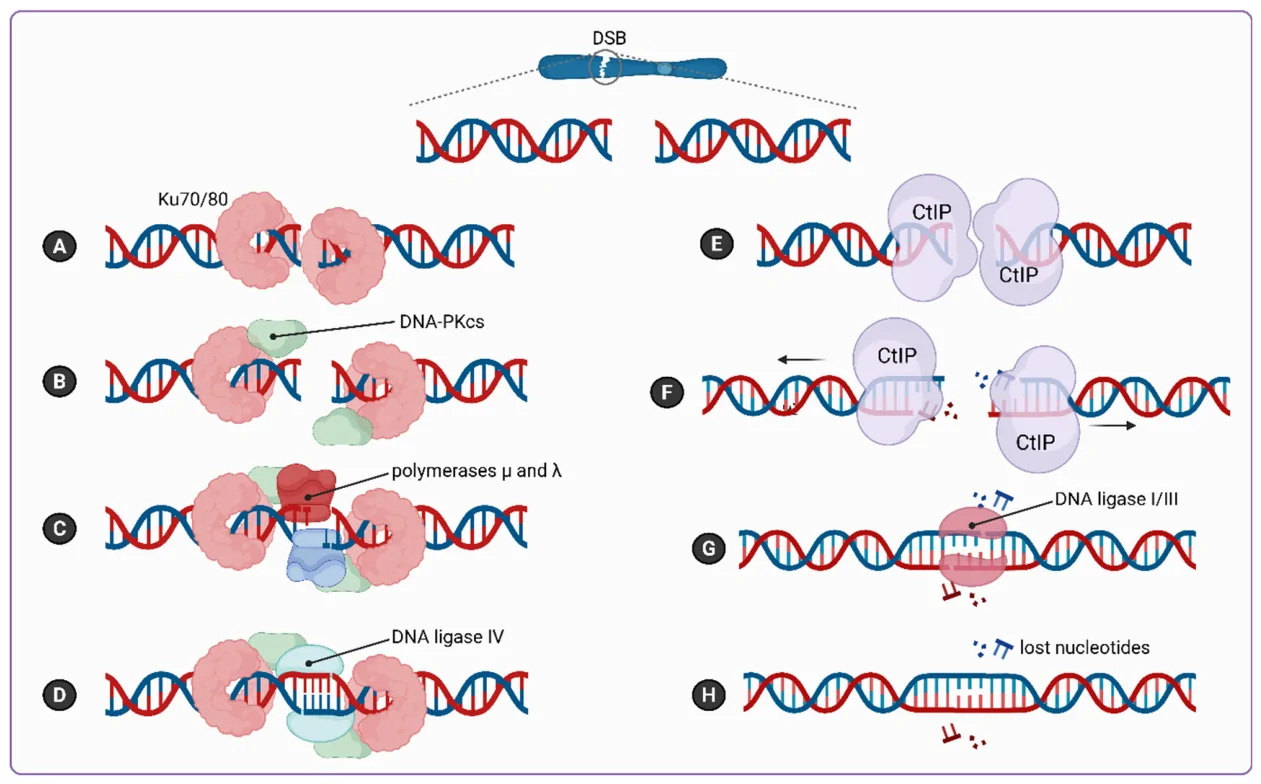
Classical non-homologous end joining (NHEJ) (**A**–**D**) and alternative end joining/microhomology-mediated end joining (AEJ/MMEJ) (E-H) repair of DSBs. Upon NHEJ, direct ligation of broken DNA ends is facilitated by the protection of exposed DNA ends by the Ku70/80 (**A**) and DNA-PK-dependent complexes (**B**), to directly ligate free DNA ends (**C**,**D**). In contrast, AEJ/MMEJ is CtIP-dependent (**E**) and exposes microhomologies between the resected strands (**F**) to facilitate the ligation of broken phosphate-pentose backbones (**G**). Both are error-prone due to the high rate of insertions (**D**) and deletions (**H**) relative to HDR. Straight black arrows in step F indicate the direction of the movement of the enzymes depicted.

In the absence of suitable homologous templates, DSBs are believed to be primarily repaired by the non-homologous end joining repair (NHEJ) that directly ligates the broken DNA ends (Figure 2) [14,21,22,23,24]. NHEJ utilizes the Ku70/80 heterodimer to protect DNA ends from 5′ to 3′ resection and recruit the DNA-dependent protein kinase catalytic subunit (DNA-PKcs) and DNA ligase IV for direct ligation [14,24]. Although NHEJ can repair DSBs precisely if DNA ends are fully compatible, it can also result in small deletions or insertions, so is considered to be error-prone [25]. When classical NHEJ is unavailable or fails, the alternative end joining pathway (AEJ) operates (Figure 2) [24,26]. AEJ is believed to be dependent on CtIP but, unlike in HDR, in AEJ it trims strands back until it exposes few-base-long microhomologies between the resected strands [24]. This mechanism, however, typically causes deletions or templated insertion in the damaged chromatids, making AEJ more error-prone than that of the classical NHEJ. Indeed, AEJ has been implicated in the rise in pathological chromosome aberrations, particularly in neoplasms [24,27]. Apparently, the choice between the NHEJ and AEJ pathways is a matter of direct and sequential competition [12,14,23,24]. If DNA ends at the DSB locus are already resected or NHEJ fails, AEJ is favored, but if they are protected by Ku70/80 binding, the AEJ pathway is repressed [24]. Canonical RAD51-dependent somatic HDR is generally restricted to the S/G2 phases due to factors like its need for sister chromatids, the low CtIP activity before the S phase or the 53BP1-mediated protection of the DNA ends that limits strand resection in the G1 phase. Thus, its activity outside the S/G2 phases is believed to be limited to artificial settings and the replication-independent DSB mutagenesis more likely triggers NHEJ or AEJ pathways.

Independent of the existence of multiple repair mechanisms of eukaryotic cells, simultaneous DSB formation in more than one chromosome can be resolved erroneously so that DNA segments are relocated or reciprocally exchanged between non-homologous chromosomes, leading to one of the most critical chromosomal structural abnormalities, termed chromosome translocation [14,27].

## 2. Chromosomal Translocations

Since chromosomes occupy dedicated nuclear territories, which affect wild-type gene expression through enhancer–promoter interactions, transcription factor accessibility and epigenetic modifications, structural rearrangements usually result in dysregulation of both local and remote gene expression profiles that might lead to abnormal cellular functions [28,29,30,31,32,33].

**Figure 3:**
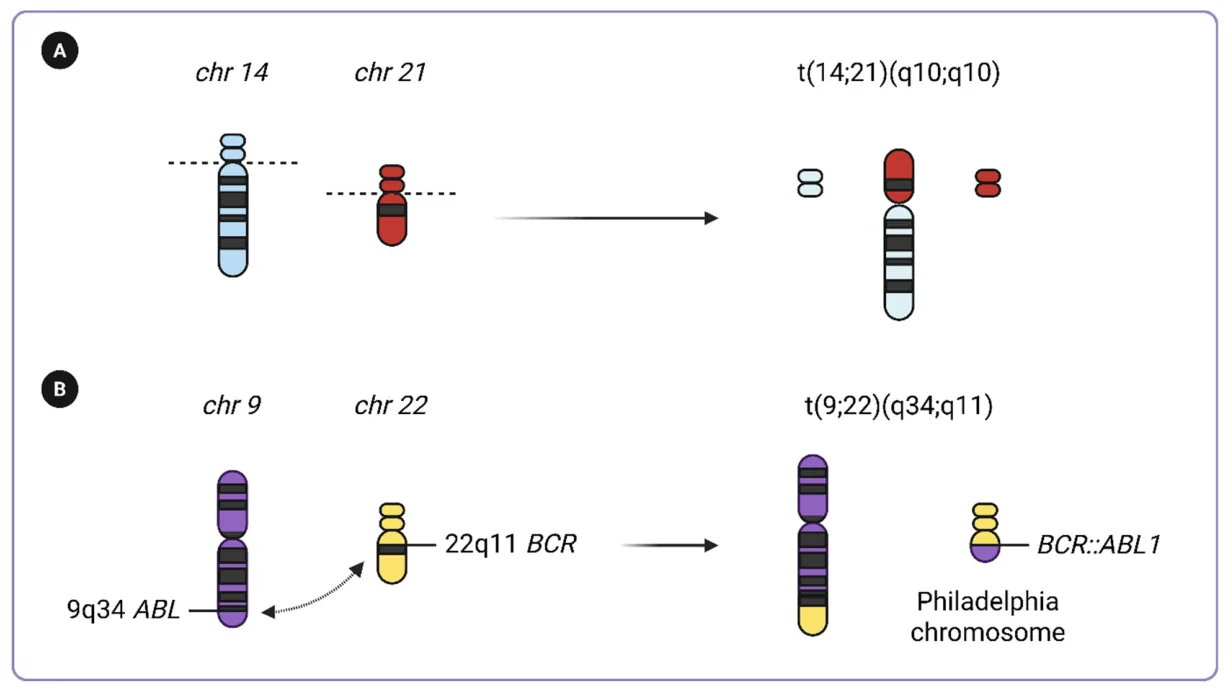
Chromosome rearrangements. (**A**) Representation of the Robertsonian translocation t(14;21)(q10;q10) showing centric fusion of the long arms of chromosome 14 and 21 with loss of their short arms, resulting in a single derivative chromosome. Dashed lines indicate the centromeric breakpoint regions of the affected chromosomes. (**B**) Ideograms of chromosomes 9 and 22 involved in the reciprocal translocation known as Philadelphia chromosome. Translocation t(9;22)(q34;11) involves chromosome regions 9q34 and 22q11 containing *ABL1* and *BCR*, respectively. During the translocation event (dashed bidirectional arrow), the 5′ portion of the *BCR* from chromosome 22 fuses with the 3′ portion of the *ABL1* from chromosome 9, giving rise to the *BCR::ABL1* fusion gene on the derivative chromosome 22 (der(22)). The reciprocal derivative chromosome (der(9)) contains the remaining exchanged genetic material. The *BCR::ABL1* fusion encodes a constitutively active tyrosine kinase that plays a central role in the pathogenesis of chronic myeloid leukemia (CML) and is also observed in a subset of acute lymphoblastic leukemias (ALLs).

Chromosomal translocations are categorized on the basis of their structural configuration and the resulting balance of the genetic material [23]. For the latter one, they can either be balanced or unbalanced depending on whether there is no net gain or loss of genetic information or the genetic information content is affected, respectively [23]. One of the most frequent balanced structural chromosomal rearrangements is the Robertsonian translocation, which occurs when two acrocentric chromosomes fuse at their centromeres while their short arms are lost (Figure 3A) [34,35,36,37]. Although they are considered functionally balanced due to the highly repetitive homologous sequences, including multiple copies of ribosomal DNA, in the short arms of acrocentric chromosomes, Robertsonian translocations cause aneuploidy, like chromosome 21 trisomy, in offspring [35,36].

Translocations involving mutual exchange of chromosomal segments between two non-homologous chromosomes producing two derivative chromosomes are termed reciprocal translocations (Figure 3B) [14,23,27,29]. Upon a non-reciprocal translocation, in contrast, a chromosomal segment becomes inserted into a non-homologous chromosome without reciprocal exchange, a phenomenon termed insertional translocation [38,39]. If a translocation disrupts coding regions, it may give rise to fusion genes or alter gene expression with often catastrophic consequences to the cell (Figure 3B) [23,24,29].

**Figure 4:**
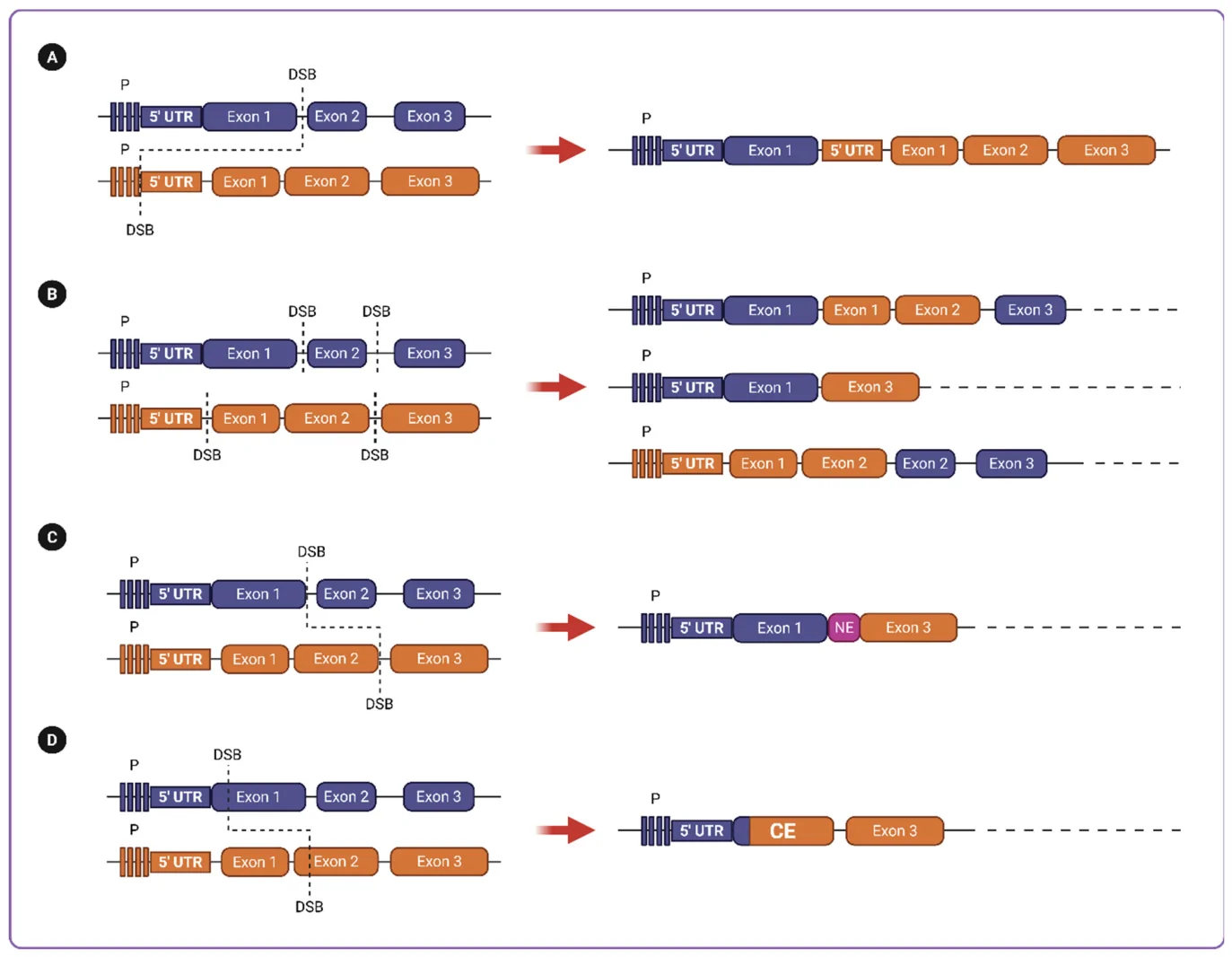
Major types of gene fusions arising from chromosomal translocations. DNA double-strand breaks (DSB) can affect both exonic and intronic sequences with differential outcomes. These include (**A**) neo-translational fusions, where one DSB hits the DNA between the promoter (P) and the downstream 5′ untranslated regions (5′UTR) so the 5′UTR becomes part of the fusion gene, (**B**) intronic versioning that may result in various fusion constructs, (**C**) formation of novel exons (NE) if DSBs disturb splice sites and (**D**) direct fusion of former exons giving rise to chimeric exons (CE).

On the mechanistic basis of their formation, chromosome translocations are categorized into several distinct classes (Figure 4) [40]. When the repair of broken DNA results in the fusion of a coding exon or exons of one gene upstream of the 5′ untranslated region (5′ UTR) of another, the original 5′ UTR of the latter gene becomes part of the coding region of the fusion gene (Figure 4A) [41]. These so-called neo-translational fusions may dictate the translation of polypeptides with novel characteristics [42]. DNA breakpoints can occur within the open reading frames of genes involved in the translocation. In some of these scenarios, fusion breakpoints can affect multiple intronic sequences leading to the formation of fusion genes with differential exon constellations, the phenomenon termed intronic versioning (Figure 4B) [43,44]. When DNA breakpoints disrupt wild-type splice sites, the splicing machinery may utilize a “neo-splice” site to compensate for a frameshift, thus ensuring the inclusion of affected exons [45]. Neo-splicing may also result in the genesis of cryptic exons, further disturbing the integrity of the encoded gene product (Figure 4C) [46]. Finally, chromosome translocations may lead to the fusion of two coding exons of distinct genes that generates chimeric exons encoding polypeptides with novel biological characteristic (Figure 4D) [47,48].

## 3. Pathogenic Translocations

Peter Nowell’s 1960 discovery of a small abnormal chromosome in chronic myeloid leukemia cells, known as the Philadelphia chromosome, marked the beginning of cancer cytogenetics, although technology at the time did not allow identification of the detailed nature of the abnormality (Figure 3B) [49]. Janet Rowley’s development of chromosome banding techniques in the early 1970s enabled her to identify the Philadelphia chromosome as t(9;22), establishing that specific translocations could define cancer subtypes [50,51]. Following this breakthrough, researchers rapidly identified numerous other cancer-related translocations. The t(15;17) in acute promyelocytic leukemia, the t(8;14) in Burkitt lymphoma, and many others were characterized cytogenetically through the 1970s and 1980s [52,53,54,55,56]. Molecular cloning in the 1980s and 1990s, then, identified the genes involved in those translocations and their related fusion proteins [57]. These efforts revealed that the t(9;22)(q34;q11) Philadelphia chromosome fuses *BCR* of chromosome 22 and *ABL1* of chromosome 9, giving rise to the *BCR::ABL1* fusion oncogene [50,51]. The translated chimeric protein is a constitutively active tyrosine kinase that drives the formation of chronic myeloid (CML) and some acute lymphoblastic leukemias [58]. Understanding molecular details of the Philadelphia chromosome eventually led to the development of imatinib, a specific BCR::ABL1 inhibitor, that revolutionized the clinical treatment of CML and demonstrated the power of targeting translocation-generated oncogenes [58,59]. Indeed, targeting the fusion protein of the t(15;17) translocation in acute promyelocytic leukemia (APL) ended in a similarly remarkable success story. t(15;17) in APL fuses *PML* with *RARA*, creating an oncogenic transcription factor that blocks myeloid differentiation [60,61]. The all-trans retinoic acid (ATRA) and arsenic trioxide, however, specifically target the PML::RARA fusion protein, leading to its degradation, allowing normal differentiation of the lymphocytes to proceed [62,63,64,65,66]. These examples highlight the fundamental importance of understanding the biology of tumor-driver translocations to facilitate the development of targeted cures for related human pathologies. These efforts, however, require reliable in vitro modeling of chromosome translocations that have presented a genuine experimental challenge so far. Aberrant functions of gene products created by translocations, particularly if involved genes encode transcription factors or tyrosine kinases, may disturb cell cycle control, giving rise to oncogenic factors (Supplementary Tables S1 and S2).

## 4. Molecular Drivers of Chromosome Translocations

The frequency and specificity of translocations is influenced by several factors [40]. Spatial proximity of chromosomes within the nucleus strongly affects which chromosomes can participate in translocations [29,34]. Chromosomes or regions that occupy nearby chromosome territories are more likely to be involved in translocations when DSBs occur [14,34,37]. The status of chromatin also affects both DSB formation and repair pathway choice since the euchromatin is generally more prone to damages [67,68]. While gene-dense euchromatic chromosomes are typically located toward the nuclear interior, and gene-poor *loci* associated with heterochromatin are often found at the nuclear periphery, chromosome translocations can induce reorganization of the nucleus, shifting chromosomes from the periphery to a more central, active position, or vice versa, which, in turn, alters the anomalous chromatin environment and gene expression [29].

Another critical factor of the translocation probability is the DNA sequence context at breakpoint regions, as certain sequences are more prone to DNA breaks than others [23,24,34,37]. DNA architectural features and repeat-rich regions, like the satellite III subfamilies on acrocentric chromosomes, result in heightened susceptibility to rearrangements [34,37]. These repetitive sequences can produce unusual DNA structures that arrest or stall replication forks, directly leading to the formation of DSBs [34,37]. Indeed, genomic *loci* with short, 4–6 bp long homologous motifs, like the GGAAT monomer found in satellite III subfamilies, exhibit elevated DNA strand-break frequency [34,37]. Accordingly, clustered distribution of breakpoints in fusions like *TCF3::PBX1* indicates that at least some rearrangements are facilitated by local DNA characteristics rather than random DNA breaks [40].

Since genesis of chromosome translocations requires two simultaneous DSBs on different chromosomes and their subsequent erroneous ligation upon the DSB repair, the cell cycle also influences the nature of rearrangements by activating distinct repair pathways [13,14,21,40,69]. One of the key mediators of the cell cycle-dependency of DSB repair is the Cyclin-dependent kinase 1 (CDK1) that links cell cycle regulation to DNA damage response [13,70]. Once cells enter the S/G2 phase, activated CDK1 mediates activating phosphorylation of the endonucleases CtIP and DNA2 for the 5′ to 3′ resection of free DNA ends at DSB *loci* [13,16]. This allows the use of homologous templates for high-fidelity repair of the damaged chromosome during the specific kinetic window of the S and G2 phase. In the G1 phase, in contrast, the absent CDK1 activity fails to promote HDR, causing compensatory increase in the more error-prone non-homologous end joining repair activity that typically results in deletions or insertions in the DSB locus [13]. It is noteworthy, however, that if non-allelic *loci*, like repetitive elements, are erroneously used as repair templates, HDR can also mediate chromosome aberrations including translocations [13].

## 5. Experimental Approach of Chromosome Translocations

Understanding the physiologic consequences of chromosome translocations is essential for developing therapeutic modalities for related pathologies. Although existing established cell lines often harbor chromosome rearrangements, aggregated mutations make dissection of their standalone effects challenging in these model systems. To address these issues, in vitro model systems are needed where, ideally, chromosome translocations are induced as defined rearrangements at endogenous *loci* while preserving native regulatory elements. Although a wide range of stimuli, including ionizing radiation, laser microirradiation, radiomimetic compounds and topoisomerase inhibitors, are known to induce chromosome translocations, most of them are not specific enough to be useful for establishing in vitro models for chromosome rearrangements. Indeed, ionizing radiation is a well-known inducer of both DSBs and SSBs, but ionizing radiation-induced DNA damages are stochastic and usually accompanied by base alterations resulting in heterogeneous lesions at uncontrolled *loci* [71,72]. Laser microirradiation is a more controlled way to induce DNA damages, however, by inducing a large number of clustered DNA lesions along the light beam path, this method still cannot achieve spatial specificity [73]. The use of genotoxic compounds, like radiomimetic drugs or topoisomerase inhibitors, has similar disadvantages, since their effects are not restricted to predetermined genomic *loci* either [74,75,76].

The first in vitro induction of non-random chromosome translocations was achieved by the use of a modified yeast mitochondrial intron-encoded meganuclease termed I-*Sce*I [77]. It belongs to the family of site-specific endonucleases that recognize long, typically 12–40 bp DNA sequences, hence the name “meganucleases”, and generate DSBs. Expression of I-*Sce*I, that has an 18 bp recognition sequence, allowed the induction of DSBs and initiation of HDR in mammalian systems [78,79]. Although historically I-*Sce*I became the most commonly used one, other meganucleases have also been developed to induce programmed DSBs. Some of them have been used by exploiting their natural affinity to specific *loci*, like I-*Ppo*I for ribosomal-DNA regions; others, like pI-*Sce*I, I-*Cre*I or *Asi*SI, have been engineered to modify or widen their sequence specificity. Indeed, engineered derivatives of I-*Cre*I, and less frequently pI-*Sce*I, have been used to induce DSBs at targeted *loci*, while *Asi*SI-estrogen receptor fusions allowed the inducible formation of DSBs upon 4-hydroxytamoxifen exposure [80,81,82]. While these were useful experimental tools for studying recombination or HDR, they turned out to be less practical for initiating chromosome rearrangements in an efficient and freely modifiable manner, partly due to their rare-cutting nature and partly because, upon cleavage, they usually generate 3′ overhanging strands that bias repair toward homology-based processes.

**Figure 5:**
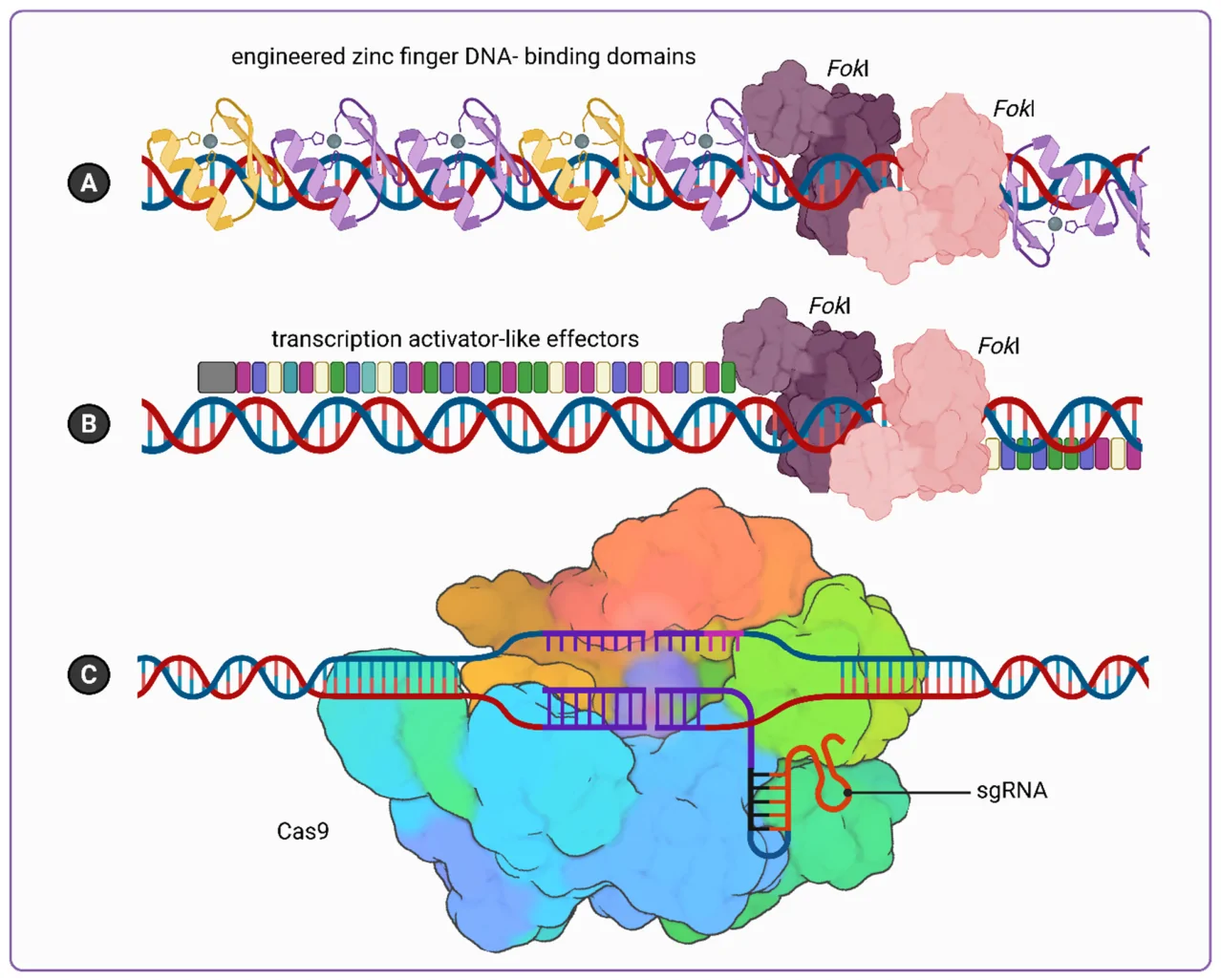
Comparison of programmable genome-editing nucleases. Schematic representation of the three major genome-editing platforms: zinc finger nucleases (**A**), transcription activator-like effector nucleases (**B**), and CRISPR/Cas9 (**C**). ZFNs and TALENs use engineered DNA-binding protein domains fused to the *Fok*I nuclease to recognize specific DNA sequences and generate double-strand breaks. Differentially colored alpha-helices (**A**) and blocks (**B**) represent the sequence- and nitrogenous base-specific domains of ZFNs and TALENs, respectively. In contrast, CRISPR/Cas9 uses a single-guide RNA (sgRNA) to direct the Cas9 nuclease to a complementary DNA target sequence, where Cas9 catalyzes symmetrical break of both strands of the targeted double-stranded DNA.

The next step forward was the development of programmable zinc finger nucleases (ZFNs) (Figure 5) [83]. They consist of engineered zinc finger DNA-binding domains fused to the nuclease domain of *Fok*I [84]. The native *Fok*I is a so-called type IIS endonuclease that recognizes the GGATG sequence by its N-terminal DNA-binding domain and cleaves DNA outside of the recognition site. Cleavage is catalyzed by its C-terminal region on both DNA strands and the two cuts are offset by 9 and 13 nucleotides, producing a 4-nucleotide 5′ overhang [85]. The modular structure of *Fok*I allowed the replacement of its N-terminal DNA-binding domain with engineered zinc finger DNA-binding domains recognizing distinct cognate sequences to initiate DNA cleavage at desired *loci* [86]. Because *Fok*I functions as a dimer, ZFNs are designed to bind sequences on both sides of the locus to be cut in opposite orientation, allowing *Fok*l dimerization and initiation of DSB upon zinc finger domains-mediated DNA binding [84]. Each zinc finger DNA-binding domain recognizes a 3-base-long DNA sequence, so in ZFNs, multiple zinc finger domains, usually three to six, are combined to create proteins recognizing longer sequences [83].

Early experiments employing ZFN constructs to induce pathological chromosome rearrangements, however, shed light on further technical challenges as well. Cleaving the *MLL* breakpoint-cluster region in human lymphoblasts to model rearrangements associated with acute myeloid leukemia successfully fragmented the target *locus*, inducing small deletions, insertions, and substitutions, but apparently failed to produce the desired leukemogenic translocations, raising the need for further requirements including a second compatible break, appropriate end joining or suitable chromatin context [87]. Subsequent studies supported these predictions, since co-expression of ZFN constructs designed to target *p84* on chromosome 19 and *EWSR1* on chromosome 22 have been reported to result in reciprocal translocations between the targeted *loci* [88]. Similarly, the Ewing sarcoma-associated t(11;22)(q24;q12) was also successfully induced by simultaneous cleavage of endogenous breakpoint regions at the target *loci* using paired ZFN constructs targeting *FLI1* and *EWSR1* on chromosome 11 and 22, respectively, [31]. Analyses of the newly formed junctions revealed small deletions, microhomologies, and occasional complex insertions, characteristics of breakpoints in patient-isolated tumors indicating that the method is capable of inducing chromosome translocations modeling existing human pathologies [31]. In addition, successful combination of I-*Sce*I with a ZFN construct targeting an I-*Sce*I consensus sequence on chromosome 6 and the *IL2RG* on the X chromosome demonstrated the complementary nature of the ZFN and meganuclease approaches, widening their standalone versatility [30]. Similar to meganucleases, however, the disadvantage of ZFN-based initiation of artificial DSBs is that each new target sequence requires the engineering and validation of novel zinc finger combinations that limited the widespread adoption of the method in everyday laboratory practice [84].

The development of transcription activator-like effector nucleases (TALENs) aimed to solve the limitations of ZFNs by using simpler DNA-binding domains derived from plant pathogenic bacteria like the *Xanthomonas campestris* pv. *vesicatoria* (Figure 5) [89,90,91]. Analyses of their virulence led to the discovery of the *avrBs3* gene that contains a region of seventeen, highly conserved, tandemly repeated, 102 bp long sections and encodes a DNA-binding protein, termed the transcription activator-like effector (TALE) [91]. Its sequence specificity is mediated by its repeated 34-amino-acid-long domains, each recognizing a single DNA base by their repeat-variable residues at position 12 and 13 named repeat-variable diresidues (RVD) [92,93]. Fusion of the TALEs DNA-binding domains with *Fok*I led to the development of a novel class of programmable endonucleases called TALENs [89]. The modular structure of their DNA-binding domain and the single-base recognition capacity of RVDs allow them, in principle, to target almost any genomic location offering greater freedom in target choice compared to meganucleases or ZFNs [89]. Since TALENs use *Fok*I nuclease domains to cleave DNA strands, they also need to work in pairs, so their dimeric structure follows the logic of ZFNs (Figure 5) [84,89].

Similar to ZFNs, TALENs were successfully used to generate several cancer-related chromosome translocations [90,94,95]. These include early studies on the induction of t(2;5)(p23;q35) that fuse *NPM1* and *ALK* and are associated with anaplastic large-cell lymphoma (ALCL) and the reciprocal t(9;11) translocation in human cord-blood CD34^+^ cells [96]. These studies also demonstrated that chromosome translocations can be induced by the programmable endonucleases without first inserting artificial break sites into the participating chromosomes [96]. In addition, although exposure to TALEN induced translocation in a minority of the cells only, transplanting them into mice led to the development of myeloid leukemia with patient-like molecular and pathological features, suggesting that the model systems created by the use of site-directed endonucleases can functionally be equivalent to the naturally occurring pathogenic chromosome translocations [96].

Although both platforms use the strategy that programmable DNA-binding proteins position dimeric *Fok*I domains exploiting protein–DNA recognition for locus targeting, they have distinct disadvantages. While ZFNs are relatively compact, making transfection of their encoding constructs more feasible, retargeting requires extensive and often challenging protein engineering. In contrast, although the modular structure of their DNA-binding domains makes TALENs more flexible upon retargeting, they require cloning of long repetitive DNA sequences that makes the design and generation of the large recombinant construct to be transfectable both time-consuming and technically challenging [89]. The widespread implementation of the clustered regularly interspaced short palindromic repeats (CRISPR/Cas9)-based endonuclease machinery in genome editing, apparently, provided new tools to address these issues.

Cas9 is a dual-RNA-guided DNA endonuclease that is able to cleave DNA at a targeted locus [97]. Unlike in ZFNs and TALENs, however, *loci* are targeted on the basis of complementary base pairing between the target sequence and the Cas endonucleases’ own single-stranded RNA component instead of protein–DNA interactions. Naturally, maturation of the targeting RNA (crRNA) element is mediated by RNAse III that is recruited to the crRNA by a separate, trans-encoded small RNA that has a 24-nucleotide complementarity to the repeat regions of crRNA precursor transcripts [98]. This dual RNA system has been simplified successfully into a single chimeric RNA species known as single-guide RNA (sgRNA) [99]. It has also been revealed that the targeting RNA component of these endonucleases directs them to a complementary *locus* next to a short sequence termed proto-spacer adjacent motif (PAM) [100]. The nuclease usually requires both for its full activity when the HNH domain cuts the DNA strand complementary to the sgRNA while the RuvC domain cuts the opposite strand (Figure 5) [99].

Since its target specificity is determined by the sgRNA sequence that can be more easily modified than DNA-binding proteins, the CRISPR/Cas9 system became a powerful tool for genome editing in a wide range of biological scenarios [90,95,101,102]. Further studies, however, also revealed that Cas9 specificity is not exclusively determined by the complementarity of the sgRNA [103]. Indeed, Cas9-mediated cleavages can occur even in the presence of mismatches between the sgRNA and the host DNA, revealing that off-target DNA breaks and mismatch position, sequence context, PAM identity, and nuclease dosage all influence endonuclease activity [104].

The use of the CRISPR/Cas9 strategy in the induction of chromosome translocations involves transfection of cells with two distinct guide RNAs, each targeting different *loci* on separate chromosomes [95,101,102]. The idea is that, upon its sgRNA-guided binding to the DNA, co-transfected Cas9 creates DSBs at both sgRNA-targeted sites and, upon their NHEJ-mediated repair, the desired translocation of the targeted chromosomes may occur [90,95]. This approach has been successfully used to generate numerous cancer-related translocations, including *BCR::ABL1*, *EWSR1::FLI1*, and *PML::RARA,* allowing detailed study of their biological effects [94,95,105]. The early use of the CRIPR/Cas9 system to induce cancer-associated chromosomal translocations also demonstrated that the desired rearrangements occur three to four log-units more frequently than upon the use of other nucleases or recombinases [95]. In contrast, PAM availability and orientation could still constrain Cas9 targeting even when the intended breakpoint sequence is otherwise suitable [99]. These issues fueled a widespread search for variants showing greater PAM-flexibility, reduced off-target activity, increased specificity, and easier transfectability, leading to the identification of mutants that, similar to ZFNs or TALENs, act like paired nickases, as well as Cas9 variants that are substantially smaller than their *S. pyogenes* counterpart, allowing their successful viral delivery into mammalian cells in vivo [106,107,108,109,110]. By 2020, these efforts led to a catalog of more than 70 Cas9 orthologs, each with distinct biochemical characteristics, while a recent metagenomic analysis shifted Cas9 discovery further to computationally mapping tens of thousands of candidates by predicting their PAM preferences [111,112].

Although the CRISPR/Cas9 technique rapidly became the preferred tool for most genome editing applications, the efficiency of CRISPR/Cas9-mediated generation of translocations varies depending on multiple factors, including the characteristics of target *loci*, the distance between targets, the delivery method, nuclease activity and the probability that two broken chromosome ends encounter and are repaired with one another [101,102]. These challenges fueled further developments of genome editing tools, leading to methods like the prime editor 2 nuclease-mediated translocation and inversion (PETI) [113]. Prime editor 2 (PE) is a fusion nuclease of a Cas9 nickase and a reverse transcriptase (RT) loaded with an RNA (pegRNA) that serves both as a guide RNA and as a template for the Cas9 and the RT components, respectively [114]. In the presence of two pegRNAs, it is assumed that complementary 3′ DNA flaps of the opposite target sites facilitate the rejoin of otherwise non-related chromosome ends that allows programmed non-homologous translocations [115]. Indeed, PETI has successfully been used to generate cancer-associated rearrangements including *NPM1::ALK* and *KIF5B::ALK* translocations [114,115]. PETI was demonstrated to increase the proportion of junctions with the intended sequence configuration and reduced random junctional modifications compared with conventional Cas9. However, since its performance depends on pegRNA sequence, flap design, target orientation and locus-specific repair activity, in its current form, PETI cannot completely eliminate DSB-associated repair errors, and indels, incomplete reciprocal products, unbalanced rearrangements or unintended structural variants can still occur [115].

TIGR-Tas represents a potentially useful addition to the Cas9-based genome-engineering toolkit because it allows PAM-independent DNA targeting [110]. TIGR-Tas systems use tandem interspaced guide RNAs that are processed into 36-nucleotide-long RNAs (tigRNAs), which guide the Tas protein to specific DNA sequences. A key distinction from the CRISPR/Cas9 system is that tigRNAs contain dual spacers that recognize both DNA strands and they do not require a PAM sequence, which increases flexibility and specificity simultaneously, although its relevance to chromosome translocation modeling in human systems remains to be established.

Another recent improvement of the Cas9-based methods is deterring the NHEJ pathway to homologous end joining repair by the combinatorial use of directed paired Cas9 nickases, with additional donor molecules homologous to both of the chromosome ends to be fused for the desired heterogenous translocation, facilitating the spatial alignment of broken non-related chromosome regions [116]. Modeling the *BCR::ABL1* and *EML4::ALK* translocations, the donor-assisted homologous recombination-mediated translocation was showing not only dramatically increased translocation frequencies but reduced the occurrence of unwanted indels, substantially improving precision in the genesis of programmed rearrangements [116].

Thus, no single platform is optimal for all translocation-modeling experiments. ZFNs and TALENs may remain useful when protein-based targeting, compact constructs or performance in particular primary-cell systems are advantageous. In contrast, CRISPR/Cas9 is generally the most accessible and multiplexable platform, whereas PETI may be preferable when a more defined junction is required, indicating that platform selection should consider the desired rearrangement, target-site architecture, cell type, delivery method, expected repair pathway and the level of structural-variant validation required (Table 1) [89,94].

Regardless of the genome-engineering platform used, thorough, multiplex analyses of the intended translocation is critical. Indeed, short-range PCR and amplicon sequencing can confirm expected breakpoints but may miss large deletions, inversions, loss of heterozygosity, interchromosomal rearrangements and complex on-target structures extending beyond the assayed region [117]. Duplicated or inverted fragments, exogenous DNA integration and clustered rearrangements can also apparently occur at correctly edited *loci*, but these alterations, while they can affect cell survival, may interfere with the disease-specific phenotype. Thus, one can speculate that targeted long-read sequencing of the edited genome is inevitable upon chromosome translocation modeling [118]. Indeed, methods like the single targeted linker-mediated PCR sequencing (CAST-Seq) can provide a sensitive approach for detecting and quantifying unwanted chromosomal rearrangements, but, if they rely on the use of targeted primers for library construction steps, may miss rearrangements that do not involve the selected *locus* [119]. Thus, combinations of distinct, complementing methods like copy-number and allele-balance assessments, targeted long-read sequencing, unidirectional targeted sequencing, or cytogenetic methods seem to be inevitable for appropriate validation of chromosome translocation models [120].

## 6. Conclusions and Future Perspectives

Development of chromosome banding techniques in the late 1960s and early 1970s revolutionized cytogenetics. Indeed, quinacrine mustard and Giemsa staining became the standard methods for the detection of structural chromosome abnormalities, including translocations, with resolutions of up to approximately 5–10 megabases [121,122,123]. These techniques allowed surprisingly precise karyotyping by the visualization of complete chromosome sets of cells arrested in metaphase when chromosomes are maximally condensed [50,57]. These analytic methods accelerate the detection of the pathognomonic DNA aberrations, facilitating the design of targeted therapeutic approaches. The latter, however, has been long hindered by the lack of appropriate in vitro model systems of chromosome translocations, calling for the development of methodologies by which scientists can generate translocations experimentally according to their needs.

This challenge has been addressed by the development of techniques from the early use of meganucleases through the tailored platforms of ZFNs and TALENs, to the recent CRISPR-Cas9 revolution that provided even more powerful platforms for modeling chromosome translocations. Despite the continuous development, like the obligate-heterodimeric *Fok*I variants or improved sgRNA design and high-fidelity Cas9 variants, however, off-target cleavage and unintended rearrangements remained the most significant challenge for engineered genome editing [124,125]. Recent advances in technologies address different aspects of the problem of uncontrolled rearrangements rather than providing a single universally optimal solution. Engineered Cas variants can improve breakpoint accessibility or reduce unintended cleavage, whereas PETI improves junctional control. A method that reproduces heterogeneous NHEJ-mediated junctions may be preferable for studying translocation formation, whereas a donor-guided or prime-editor-assisted strategy combined with suppression of the NHEJ pathway may be preferable when a defined breakpoint is required [116]. Introduction of promising new techniques, including the TIGR-Tas and related RNA-guided systems may further expand programmable chromosome engineering in the future [110]. These methods should, therefore, be selected according to the experimental objectives; conventional NHEJ-based systems may better reproduce the heterogeneous junctions of naturally arising translocations, while prime-editor-assisted systems are more suitable when defined breakpoint sequences are required.

Choosing the most suitable methods for inducing the designed DNA DSBs, however, is not the only aspect that requires careful consideration. Indeed, artificial genesis of *EWSR1::CREB1* and *EWSR1::ATF1* fusions using CRISPR/Cas9 in human embryonic stem cells turned out to be lethal in undifferentiated species but not in ones differentiated into mesenchymal progenitors where they induced phenotypic traits of angiomatoid fibrous histiocytoma and gastrointestinal clear cell sarcoma, clearly indicating that cell types and the cellular status are also critical determinants for a useful model system [126]. These challenges may be addressed by using donor molecules homologous to both targeted DSBs where the donor can not only define the rearranged junction but also incorporate a selectable gene-trap cassette, allowing both the easier isolation of rare translocation-bearing cells and the conditionally induction of the resulting fusion gene [102]. This approach can be particularly useful when the translocation itself does not provide a strong selective advantage or when fusion-protein expression would interfere with the survival of the newly edited cells. Another particularly relevant aspect of the chosen cellular context is that the diverse structural outcomes of genome editing are often undetectable by standard, short-range indel analysis of the targeted *loci*, leading to an underestimation of genomic damage [119]. In primary cells, where the intact p53 status allows the p53-mediated DNA damage response, large numbers of unwanted DNA lesions can further reduce the persistence of rarely occurring desired chromosome rearrangements [127,128]. Thus, besides the thorough consideration of the most appropriate cellular model, careful selection of methods suitable for genome-wide analyses must also be an important part of the design process of in vitro models of chromosome translocations.

Considering its clinical benefits, the importance of in vitro modeling of chromosome translocations is unquestionable. Identification and understanding of fusion tyrosine kinases have directly informed the development of fusion tyrosine kinase inhibitors, like imatinib for BCR::ABL1, crizotinib for ALK and ROS1 chimeras, and larotrectinib for aberrant NTRK fusions, leading to improved clinical responses during their use in related human pathologies [129]. These achievements clearly demonstrate the urgent need for precise modeling of chromosomal rearrangements in order to expand our portfolio of novel, engineered therapeutic agents against the deleterious effects of pathological chromosome translocations.

## Figures and Tables

**Table 1:** Comparison of programmable genome-engineering platforms for modeling chromosome translocations and other structural rearrangements. This table summarizes the principal genome-engineering platforms used or proposed for modeling chromosome translocations. These approaches differ in targeting mechanism, cleavage geometry, retargeting flexibility, construct size, and control over breakpoint repair.

Platform	Main Advantages	Main Limitations	Design Constraints	Best Experimental Use
Meganucleases	long recognition sites can provide high target specificity some systems are inducible useful for controlled DSB and HDR studies	limited target choice difficult retargeting cleavage often produces 3′ overhangs less flexible for two independent breaks	requires suitable long recognition sequences or an engineered nuclease variant each new target requires a new protein-design and validation	proof-of-principle studies of DSB repair, recombination and HDR
ZFNs	compact protein constructs no CRISPR-style PAM requirement	each new target requires protein engineering and validation target activity can be context-dependent possible recleavage	compatible triplet combinations two binding sites flanking the intended break suitable spacer length correct orientation for FokI dimerization	modeling endogenous translocations
TALENs	simple targeting code broad target-site flexibility no CRISPR-style PAM requirement	time-consuming assembly two proteins are required challenging cloning and transfection	TALE array dependence spacer length and orientation	modeling specific translocations when target-site flexibility is more important than construct size
CRISPR/Cas9	simple retargeting inexpensive guide design highly multiplexable high rearrangement efficiency suitable for screening multiple *loci*	PAM dependence off-target cleavage locus- and delivery-dependent efficiency indels, unbalanced products and complex junctions possible p53-mediated toxicity in primary cells	requires a suitable guide sequence, PAM dependency two compatible guides must be identified at separate *loci*	routine generation of known cancer-associated translocations and inversions high-throughput screening of rearrangement mechanisms
Prime-editor/PETI	more defined rearrangement junctions reduced random indels and complex rearrangements	DSBs induction locus-specific pegRNA design incomplete reciprocal products, indels and unintended structural variants complex delivery	requires suitable Cas9 target sites PAM dependent nicking geometry, two pegRNAs, compatible reverse-transcription templates complementary to the 3′ flap sequences	modeling defined translocations or inversions when junction configuration and sequence precision are especially important
TIGR-Tas	very small effector PAM-independent targeting useful for delivery-constrained applications unusual dual-strand targeting mechanism	less mature than Cas9 limited activity in human cells locus-dependent chromosome translocations and inversions have not yet been demonstrated	targetability depends on tigRNA architecture, dual-spacer sequence compatibility, guide maturation and the biochemical properties of the chosen Tas ortholog	a proof-of-principle platform for programmable cleavage where PAM flexibility or compact delivery is important

## Data Availability

No new data were created or analyzed in this study. Data sharing is not applicable to this article.

## Supplementary Materials

The following supporting information can be downloaded at https://www.mdpi.com/article/10.3390/cimb48101032/s1 [130,131,132,133,134,135,136,137,138,139,140,141,142,143,144,145,146,147,148,149,150,151,152,153,154,155,156,157,158,159,160,161,162,163,164,165,166,167,168,169,170,171,172,173,174,175,176,177,178,179,180,181,182,183,184,185,186,187,188,189,190,191,192,193,194,195,196,197,198,199,200,201,202,203,204,205,206,207,208,209,210,211,212,213,214,215,216,217,218,219,220,221,222].

## Abbreviations

ABL1	ABL (Abelson murine leukemia virus) proto-oncogene 1, non-receptor tyrosine kinase
AEJ	Alternative end joining
ALK	Anaplastic lymphoma kinase
ALL	Acute lymphoblastic leukemia
APL	Acute promyelocytic leukemia
BCR	Breakpoint-cluster region protein
BRCA1	Breast cancer type 1 susceptibility protein
CDK1	Cyclin-dependent kinase 1
CML	Chronic myeloid leukemia
CRISPR	Clustered regularly interspaced short palindromic repeats
CtIP	C-terminal binding protein interacting protein
DMC1	DNA meiotic recombinase 1
DNA-PKcs	DNA-dependent protein kinase catalytic subunit
DSB	Double-strand break
dHJ	Double Holliday junction
EWSR1	EWS RNA binding protein 1
FLI1	Fli-1 proto-oncogene, ETS transcription factor
FokI	Flavobacterium okeanokoites restriction endonuclease I
HDR	Homology-directed repair
HJ	Holliday junction
HNH	Histidine–asparagine–histidine nuclease domain
KIF5B	Kinesin family member 5B
MMEJ	Microhomology-mediated end joining
MRE11	MRE11 homolog, double-strand break repair nuclease
NHEJ	Non-homologous end joining
NPM1	Nucleophosmin 1
PALB2	Partner and localizer of BRCA2
PAM	Proto-spacer adjacent motif
PETI	Prime editor nuclease-mediated translocation and inversion
PML	Progressive multifocal leukoencephalopathy
ROS1	ROS proto-oncogene 1, receptor tyrosine kinase
RPA	Replication protein A
RVD	Repeat-variable diresidue
SC	Synaptonemal complex
sgRNA	Single-guide RNA
SPO11	SPO11 initiator of meiotic double-strand breaks
SSB	Single-strand break
TALE	Transcription activator-like effector
TALEN	Transcription activator-like effector nuclease
Tas	TIGR-associated protein
TCF3	Transcription factor 3
TIGR	Tandem interspaced guide RNA
tigRNA	TIGR-derived guide RNA
UTR	Untranslated region
WT-PE	Wild-type prime editor
ZFN	Zinc finger nuclease
