# Supplementary material for: In Vitro Chromosome Translocations: Modeling Critical Drivers of Human Neoplasms
Source: Curr Issues Mol Biol. 2026 Oct 4;48(10):1032. doi: 10.3390/cimb48101032 (PMC13703145; doi:10.3390/cimb48101032)
Supplement: Supplementary file 1 [file cimb-48-01032-s001.zip › cimb-4533089-supplementary.pdf]

Supplementary Materials

In vitro chromosome translocations: modelling critical drivers of human neoplasms

Nuray Tynyshtykbayeva [1], Kamilla Varga [3], Szilvia Lukacsi [3], Zsolt Fabian [2, 3 and *]

[1]Institute of Biology, Faculty of Science, Eötvös Loránd University, 1 Pázmány Péter sétány, Budapest, 1117 Hungary
[2]School of Medicine and Dentistry, Faculty of Clinical and Biomedical Sciences, University of Lancashire, Fylde Rd, Preston PR1 2HE, UK
[3]Translocon Biotechnologies plc, 6. Akadémia str., Budapest, 1054 Hungary
* Correspondence: zfabian@lancashire.ac.uk

current issues in molecular biology

MDPI

Table S1. Chromosome translocations resulting in fusion tyrosine kinases. Fusion tyrosine kinases-generating chromosomal translocations. To generate a functional fusion polypeptide, breakpoints typically occur within intronic regions facilitating in-frame mutations. They recurrently occur in genes, like *ETV6*, *BCR*, or *KIF5B*, that are involved in fusions with multiple tyrosine kinase domains. Other fusions preferentially occur at *loci* that correlate with protein stability and oncogenic potential. For example, *EML4::ALK* fusions in NSCLC predominantly involve *EML4* exon 13 or 20 fused to *ALK* exon 20, resulting in highly stable, oncogenic proteins. Recent advances in sequencing have uncovered rare and previously unrecognized fusion tyrosine kinases like the ETV6::SYK, ITK::SYK and ETV6::LYN with detailed molecular characterization yet to be done.

Fusion Product	Genotype	Genes, affected	Associated Disease	Ref
ANO3::FGFR4	t(2;5)(q37;q35)	*ANO3, FGFR4*	NSCLC	[131]
BCR::ABL1	t(9;22)(q34;q11)	*BCR, ABL1*	Chronic Myeloid Leukemia (CML), Acute Lymphoblastic Leukemia (ALL)	[132-135]
BCR::FGFR1	t(8;22)(p11;q11)	*BCR, FGFR1*	CML-like Myeloproliferative Disorders	[136]
BCR::RET	t(22;10)(q11;q11)	*BCR, RET*	Myeloid/Lymphoid Neoplasm with Eosinophilia	[137]
CD74::ROS1	t(5;6)(q33;q22)	*CD74, ROS1*	NSCLC	[138,139]
CLTC::SYK	inv(17)(q11;q21)	*CLTC, SYK*	Juvenile Xanthogranuloma	[140]
CNTRL::FGFR1	t(8;9)(p11;q33)	*CNTRL, FGFR1*	Myeloid/Lymphoid Neoplasms with Eosinophilia	[141]
EML4::ALK	inv(2)(p21p23)	*EML4, ALK*	Non-Small Cell Lung Cancer (NSCLC)	[142]
ETV6::ABL1	t(9;12)(q34;p13)	*ETV6, ABL1*	AML,<br>B-ALL,<br>T-ALL,<br>CML,<br>Myeloproliferative Neoplasms	[143,144]
ETV6::FGFR2	t(12;10)(p13;q26)	*ETV6, FGFR2*	Myeloid/Lymphoid Neoplasm with Eosinophilia	[145]
ETV6::FLT3	t(12;13)(p13;q12)	*ETV6, FLT3*	Myeloid/Lymphoid Neoplasm with Eosinophilia	[146]
ETV6::JAK2	t(9;12)(p24;p13)	*ETV6, JAK2, (TEL, JAK2)*	Acute Lymphoblastic Leukemia (ALL),<br>Atypical CML,	[147,148]

current issues in molecular biology

MDPI

			T-ALL	
ETV6::LYN	t(8;12)(q12.1;p13)	*ETV6, LYN*	Eosinophilic Myeloproliferative Neoplasm,<br>T-LBL,<br>AML	[149-153]
ETV6::NTRK3	t(12;15)(p13;q25)	*ETV6, NTRK3, (TEL, TRKC)*	Infantile Fibrosarcoma,<br>Congenital Mesoblastic Nephroma,<br>Secretory Breast Carcinoma,<br>AML	[154]
ETV6::PDGFRA	t(4;12)(q12;p13)	*ETV6, PDGFRA*	Myeloid/Lymphoid Neoplasm with Eosinophilia	[155]
ETV6::PDGFRB	t(5;12)(q33;p13)	*ETV6, PDGFRB, (TEL, PDGFRB)*	Chronic Myelomonocytic Leukemia (CMML),<br>Myeloproliferative Neoplasms	[156]
ETV6::SYK	t(9;12)(q22;p13)	*ETV6, SYK*	Myeloid/Lymphoid Neoplasms with Eosinophilia,<br>B-ALL,<br>Atypical MDS	[157,158]
FGFR1OP::RET	t(6;8)(q27;p11)	*FGFR1OP, RET*	Myeloid/Lymphoid Neoplasm with Eosinophilia	[137,159]
FGFR2::BICC1	inv(10)(q11.21;q22.3)	*FGFR2, BICC1*	Intrahepatic Cholangiocarcinoma	[160]
FGFR2::TACC2	t(4;10)(p16;q26)	*FGFR2, TACC2*	Intrahepatic Cholangiocarcinoma	[161]
FGFR3::ADD1	t(4;4)(p16;p16)	*FGFR3, ADD1*	Urothelial Carcinoma	[162]
FGFR3::JAKMIP1	t(4;7)(p16;q32)	*FGFR3, JAKMIP1*	Urothelial Carcinoma	[162]
FGFR3::TACC3	t(4;7)(p16;q21)	*FGFR3, TACC3*	Glioblastoma,<br>Urothelial Carcinoma,<br>NSCLC	[163]
FGFR3::TNIP2	t(4;20)(p16;q13)	*FGFR3, TNIP2*	Urothelial Carcinoma	[162]
FOP1::FGFR1	t(6;8)(q27;p11)	*FOP1, FGFR1*	Myeloid/Lymphoid Neoplasms with Eosinophilia	[141]
ITK::SYK	t(5;9)(q33;q22)	*ITK, SYK*	Peripheral T-cell Lymphoma, Follicular Helper T-cell Subtype	[164]

KIAA1549::BRAF	7q34 (tandem duplication)	*KIAA1549, BRAF*	Pilocytic Astrocytoma	[165]
KIF5B::ALK	t(2;10)(p23;q13)	*KIF5B, ALK*	Lung Adenocarcinoma,<br>ALK-positive Histiocytosis,<br>LCNEC	[166-170]
KIF5B::ALK	t(2;10)(p23;q13)	*KIF5B, ALK*	Lung Adenocarcinoma,<br>ALK-positive Histiocytosis,<br>LCNEC	[166-169,171]
NPM::ALK	t(2;5)(p23;q35)	*NPM, ALK*	Anaplastic Large-Cell Lymphoma (ALCL)	[172]
NSD1::FGFR4	t(5;5)(q35;q35)	*NSD1, FGFR4*	NSCLC	[131]
NUP214::ABL1	t(9q34;q13) (cryptic)	*NUP214, ABL1*	T-ALL,<br>B-ALL,<br>AML	[173,174]
PCM1::JAK2	t(8;9)(p22;p24)	*PCM1, JAK2*	Myelodysplastic/Myeloproliferative Neoplasms,<br>Atypical CML,<br>Secondary AML	[175,176]
PML::SYK	t(9;15)(q22;q24)	*PML, SYK*	Myeloproliferative Neoplasm,<br>AML with Fibrosis	[177]
RANBP2::ALK	t(2;2)(p23;q13)	*RANBP2, ALK*	Myeloid/Lymphoid Neoplasm with Eosinophilia	[178,179]
RET::PTC1 (CCDC6::RET)	inv(10)(q11.2;q21)	*RET, CCDC6, (RET, PTC1)*	Papillary Thyroid Carcinoma	[180]
RET::PTC3 (NCOA4::RET)	t(10;17)(q11.2;q23)	*NCOA4, RET, (RET, PTC3)*	Papillary Thyroid Carcinoma	[181]
TPM3::ALK	t(1;2)(q21;p23)	*TPM3, ALK*	Inflammatory Myofibroblastic Tumor	[182]
TPM3::NTRK1	t(1;1)(q21;q23)	*TPM3, NTRK1*	NTRK-rearranged Spindle Cell Neoplasm,<br>High-Grade Sarcoma	[183]
ZNF198::FGFR1	t(8;13)(p11;q12)	*ZNF198, FGFR1*	8p11 Myeloproliferative Syndrome (EMS)	[184,185]
ZNF346::FGFR4	t(8;8)(q24;q24)	*ZNF346, FGFR4*	NSCLC	[186]

current issues in molecular biology

MDPI

Table S2. Chromosomal translocations resulting in fusion transcription factors. List the major and clinically significant fusion transcription factors described in the literature to date. Some genes, e.g. *KMT2A*, *EWSR1*, *PAX5*, or *TFE3*, are frequently involved in these fusion events, reflecting their inherent genomic instability and potential roles in transcriptional regulation.

Fusion Product	Genotype	Genes affected	Associated Disease	Ref
ASPSCR1::TFE3	t(X;17)(p11;q25)	*ASPSCR1, TFE3*	Alveolar Soft Part Sarcoma	[187]
CRTC1::MAML2	t(11;19)(q13;p13.3)	*CRTC1, MAML2*	Mucoepidermoid Carcinoma	[188]
DEK::NUP214	t(6;9)(q22–23;p23–24)	*DEK, NUP214*	Acute Myeloid Leukemia	[188]
ETV6::RUNX1 (TEL::AML1)	t(12;21)(p13;q22)	*ETV6, RUNX1*	Pediatric B-cell ALL	[189]
EWSR1::ATF1	t(12;22)(q13;q12)	*EWSR1, ATF1*	Clear Cell Sarcoma, Angiomatoid Fibrous Histiocytoma	[190,191]
EWSR1::CREB1	t(22;2)(q12;q33)	*EWSR1, CREB1*	Angiomatoid Fibrous Histiocytoma	[192]
EWSR1::ERG	t(21;22)(q22;q12)	*EWSR1, ERG*	Ewing Sarcoma	[193]
EWSR1::FLI1	t(11;22)(q24;q12)	*EWSR1, FLI1*	Ewing Sarcoma	[194]
EWSR1::WT1	t(11;22)(p13;q12)	*EWSR1, WT1*	Desmoplastic Small Round Cell Tumor	[195]
FUS::ATF1	t(16;12)(p11;q13)	*FUS, ATF1*	Angiomatoid Fibrous Histiocytoma	[191]
FUS::DDIT3 (CHOP)	t(12;16)(q13;p11)	*FUS, DDIT3*	Myxoid Liposarcoma	[196]
KIAA1549::BRAF	t(7;17)(q34;q25)	*KIAA1549, BRAF*	Pilocytic Astrocytoma	[197]
KMT2A::AF10	t(10;11)(p12;q23)	*KMT2A, AF10*	Acute Myeloid Leukemia	[198]
KMT2A::AF6	t(6;11)(q27;q23)	*KMT2A, AF6*	Acute Myeloid Leukemia	[199]
KMT2A::AFF1	t(4;11)(q21;q23)	*KMT2A, AFF1*	Acute Lymphoid Leukemia	[200]
KMT2A::ENL	t(11;19)(q23;p13.1)	*KMT2A, ENL*	Acute Leukemia	[201]
KMT2A::MLLT1	t(11;19)(q23;p13.3)	*KMT2A, MLLT1*	Acute Myeloid Leukemia, Acute Lymphoid Leukemia	[201,202]
KMT2A::MLLT3	t(9;11)(p22;q23)	*KMT2A, MLLT3*	Acute Myeloid Leukemia	[203]
MYB::NFIB	t(6;9)(q22–23;p23–24)	*MYB, NFIB*	Adenoid Cystic Carcinoma	[204]
NAB2::STAT6	inv(12)(q13q15)	*NAB2, STAT6*	Solitary Fibrous Tumor	[205]
NUP98::NSD1	t(5;11)(q35;p15.5)	*NUP98, NSD1*	Pediatric AML	[206]
PAX3::FOXO1	t(2;13)(q35;q14)	*PAX3, FOXO1*	Alveolar Rhabdomyosarcoma	[207]
PAX5::AUTS2	t(9;7)(p13.2;q11.2)	*PAX5, AUTS2*	B-cell Acute Lymphoblastic Leukemia	[208,209]
PAX5::C20S	dic(9;20)(p13.2;q11.2)	*PAX5, C20orf112*	B-cell Acute Lymphoblastic Leukemia	[210]

PAX5::DACH2	t(9;X)(p13;q21)	*PAX5, DACH2*	B-cell Acute Lymphoblastic Leukemia	[208,211]
PAX5::ELN	t(9;12)(p13;q13)	*PAX5, ELN*	B-cell Acute Lymphoblastic Leukemia	[212]
PAX5::ETV6	t(9;12)(p13;p13)	*PAX5, ETV6*	B-cell Acute Lymphoblastic Leukemia	[210]
PAX5::FOXP1	t(9; 3)(p13;p13)	*PAX5, FOXP1*	B-cell Acute Lymphoblastic Leukemia	[208,211]
PAX5::IGH	t(9;14)(p13;q32)	*PAX5, IGH*	B-cell Non-Hodgkin's Lymphoma	[213]
PAX5::JAK2	t(9;9)(p13;p24)	*PAX5, JAK2*	B-cell Acute Lymphoblastic Leukemia	[214]
PAX5::PML	t(9;15)(p13;q22)	*PAX5, PML*	B-cell Acute Lymphoblastic Leukemia	[215]
PAX5::POM121	t(9;7)(p13.2;q11.2)	*PAX5, POM121*	B-cell Acute Lymphoblastic Leukemia	[208,216]
PAX5::ZNF521	t(9;18)(p13;q11.2)	*PAX5, ZNF521*	B-cell Acute Lymphoblastic Leukemia	[208,217]
PAX7::FOXO1	t(1;13)(p36;q14)	*PAX7, FOXO1*	Alveolar Rhabdomyosarcoma	[218]
PML::RARA	t(15;17)(q24;q21)	*PML, RARA*	Acute Promyelocytic Leukemia	[219]
PRCC::TFE3	t(X;1)(p11;q21)	*PRCC, TFE3*	Translocation-Associated Renal Cell Carcinoma	[220]
RUNX1::RUNX1T1	t(8;21)(q22;q22)	*RUNX1, RUNX1T1*	Acute Myeloid Leukemia (AML)	[221]
SS18::SSX1/2/4	t(X;18)(p11.2;q11.2)	*SS18, SSX1/2/4*	Synovial Sarcoma	[222]
TCF3::HLF	t(17;19)(q22;p13)	*TCF3, HLF*	Acute Lymphoid Leukemia	[223]
TCF3::PBX1 (E2A::PBX1)	t(1;19)(q23;p13.3)	*TCF3, PBX1*	Pre-B Acute Lymphoblastic Leukemia	[224]
WWTR1::CAMTA1	t(1;3)(p36;q25)	*WWTR1, CAMTA1*	Epithelioid Hemangioendothelioma	[225]
YAP1::TFE3	t(X;11)(p11;q22)	*YAP1, TFE3*	Epithelioid Hemangioendothelioma	[187]
